# Comparison of Classroom-Based Sedentary Time and Physical Activity in Conventional Classrooms and Open Learning Spaces Among Elementary School Students

**DOI:** 10.3389/fspor.2021.626282

**Published:** 2021-06-15

**Authors:** Jani Hartikainen, Eero A. Haapala, Anna-Maija Poikkeus, Eero Lapinkero, Arto J. Pesola, Timo Rantalainen, Arja Sääkslahti, Ying Gao, Taija Finni

**Affiliations:** ^1^Faculty of Sport and Health Sciences, University of Jyväskylä, Jyväskylä, Finland; ^2^Institute of Biomedicine, School of Medicine, University of Eastern Finland, Kuopio, Finland; ^3^Department of Teacher Education, University of Jyväskylä, Jyväskylä, Finland; ^4^Active Life Lab, South-Eastern Finland University of Applied Sciences, Mikkeli, Finland; ^5^Department of Sports Science, College of Education, Zhejiang University, Hangzhou, China

**Keywords:** sedentary behavior, breaks from sedentary time, physical activity, elementary school, classroom, open learning space

## Abstract

European children and adolescents spend most of their daily life and especially their school hours being sedentary which may increase their risk for chronic non-communicable diseases later in life. After the curriculum reform of Finnish basic education in 2014, most of the new or renovated comprehensive schools in Finland incorporate open and flexible classroom designs. Their open learning spaces may provide students opportunities to reduce sedentary behavior during school hours. Thus, waist-worn accelerometers were used to assess classroom-based sedentary time (ST), the number of breaks from sedentary time (BST), and physical activity (PA) among cross-sectional samples of 3rd and 5th grade students during two separate academic years in a school that underwent a renovation from conventional classrooms to open learning spaces. The cohort of 5th grade students before renovation had a smaller proportion of ST from total classroom time (56.97 ± 12.24%, *n* = 42 vs. 67.68 ± 5.61%, *n* = 28, mean difference = 10.71%-points, 95%CI = −15.65 to−5.77, *p* < 0.001), a greater number of BST per 60 min of classroom time (7.41 ± 1.16 breaks/h vs. 9.19 ± 1.59 breaks/h, mean difference = −1.78 breaks/h, 95%CI = −2.486 to −1.079, *p* < 0.001) and a greater proportion of light intensity PA (28.66 ± 9.99% vs. 22.56 ± 4.59%, mean difference = 6.10%, 95%CI = 2.56 to 9.64, *p* = 0.001) than the 5th grade cohort assessed after renovation. The cohort of 3rd grade student had a greater proportion of moderate-to-vigorous intensity PA (MVPA) after the renovation compared to the cohort assessed before the renovation [Mean Rank (Before) = 27.22, Mean Rank (After) = 37.58, *U* =524.0, *p* = 0.033]. Despite the greater ST found in 5th graders, schools with open learning spaces may facilitate BST or MVPA as observed in the 5th and 3rd grade cohorts in open learning spaces compared to the cohorts in conventional classrooms, respectively. Future studies should seek to investigate and develop teacher practices to capitalize the potential of open classrooms to reduce ST, since classroom renovation alone may not be a sufficient intervention as of itself. Longitudinal studies utilizing randomized controlled trials are warranted.

## Introduction

Sedentary behavior (SB) refers to any waking behavior characterized by an energy expenditure ≤ 1.5 metabolic equivalents of task, while in a sitting, reclining, or lying posture (Tremblay et al., 2017). Public health guidelines recommend that children and adolescents should limit their total sedentary time (ST), as a sedentary lifestyle may increase their risk for chronic non-communicable diseases later in life (Carson et al., 2016; Tremblay et al., 2016). Physical activity (PA) is defined as any bodily movement produced by skeletal muscles that results in energy expenditure (Caspersen et al., 1985). Sedentary bouts can be defined as a minimum period of uninterrupted sedentary time and breaks from sedentary time (BST) can be defined as a non-sedentary period in between two sedentary bouts (Altenburg and Chinapaw, 2015). Current evidence has shown positive associations between PA and physical and mental health in school-aged children, with potential positive health effects of reduced duration of sedentary bouts and increased number of BST (Janssen and LeBlanc, 2010; Healy et al., 2011; Saunders et al., 2013; Altenburg and Chinapaw, 2015; Biddle et al., 2019).

It has been estimated that children spend 40–60% of their time sedentary, which equals 5–8 hours a day (Colley et al., 2013; Ortega et al., 2013; Konstabel et al., 2014). In many Western countries, including Finland, less than half of the children and youth achieve the recommended daily 60 min of moderate-to-vigorous physical activity (MVPA) daily (Aubert et al., 2018; Bull et al., 2020). Schools can be considered as feasible locations for intervention aiming to reduce ST and increase overall PA among children because they spend a large proportion of their waking hours in school (Hegarty et al., 2016). European primary school children aged 10–12 have been shown to spend 65–70% of school time being sedentary and 5% on MVPA, boys having less ST, and more MVPA than girls (van Stralen et al., 2014; Salin et al., 2019).

Recently, general education classrooms have received more attention as possible settings to reduce ST and increase PA of students in addition to physical education classes and recess (Webster et al., 2015; Hegarty et al., 2016). In addition to studies focusing on teacher implemented PA during classroom time, some studies have focused on the role of built school environment in increasing PA and reducing ST of students (Lanningham-Foster et al., 2008; Brittin et al., 2015, 2017; Webster et al., 2015; Hinckson et al., 2016). In addition to potential health benefits of reduced ST and increased PA, increased classroom-based PA may have a positive impact on academic outcomes and students' on-task behavior (Goh et al., 2016; Watson et al., 2017).

A review of 13 studies reported that classroom design approaches reduced youth sitting time 44 to 60 min per day and increased standing time by 18 to 55 min per day during classroom time at school (Hinckson et al., 2016). Furthermore, evidence suggests that when supplemented with appropriate teaching methods, environments designed to encourage active learning increase PA levels in children compared to traditional classroom environments (Lanningham-Foster et al., 2008). Furthermore, a guideline-informed school physical environment (Brittin et al., 2015) may also decrease ST and length of sedentary bouts in children aged 8-10 years (Brittin et al., 2017). In classrooms utilizing flexible spaces including a variety of furniture and resources, adolescents were found to spend less class time sitting and accumulated more breaks in sitting, more bouts of intermittent (≤ 9 min) sitting, and fewer bouts of prolonged (≤ 30 min) sitting, than in traditionally furnished and arranged classroom when coupled with a greater use of student-centered pedagogies (Kariippanon et al., 2019).

After the curriculum reform of Finnish basic education in 2014, most of the new or renovated comprehensive schools in Finland incorporate open and flexible classroom designs and principles; the conventional self-contained classrooms are changed into more flexible, multipurpose, informal, and transformative open learning spaces (Ministry of Education Finnish National Curriculum, 2014; Niemi, 2020). These types of schools with non-partitioned instructional spaces have re-emerged as a result of educational reforms in some countries including Finland, the United Kingdom, Germany, and Spain (Mäkitalo-Siegl, 2010; Saltmarsh et al., 2015). The open learning spaces are typically equipped with moveable furniture and varying possibilities, such as privacy screens, to divide spaces (Kokko and Hirsto, 2021). Open learning spaces can take varied forms but some of the defining features include integration of physical and virtual space, multifunctionality, and affording students autonomy over their learning (Melhuish, 2011). Open learning spaces may reduce ST, increase the number of BST, and increase PA of students, as in terms of interior design, their characteristics resemble those of activity permissive classrooms including ample, multipurpose, and adaptable spaces (Brittin et al., 2015; Saltmarsh et al., 2015).

Nevertheless, the role of school indoor spaces *per se* in reducing ST, increasing number of BST, and increasing PA of students is still unclear. The studies published so far have reported use of physically active, or student-centered teaching methods accompanied with environmental renovation, or combined effects of improved indoor and outdoor facilities (Lanningham-Foster et al., 2008; Brittin et al., 2017; Kariippanon et al., 2019). There is currently, however, scant information on whether open learning spaces increase PA and reduce ST in classrooms where teaching methods are not experimentally altered. We investigated ST, BST, and PA levels among children in 3rd and 5th grades in two separate academic years before and after a school renovation into open learning spaces. We hypothesized that cohorts of 3rd and 5th grade students attending school in open learning space settings would have less ST and more BST and PA than their counterparts in the conventional classroom setting.

## Materials and Methods

### Participants and Procedures

The data in this cross-sectional case study comprise accelerometer measures drawn from two separate academic years with a total of 130 Finnish 3rd and 5th grade students in a school undergoing renovation from conventional classrooms to open learning spaces. Complete accelerometer data were obtained from 41 3rd and 42 5th grade students before renovation. After renovation data were obtained from 19 3rd and 28 5th grade students. In the Finnish school system 3rd graders are usually 9-years and 5th graders are 11-years of age.

**Figure 1:**
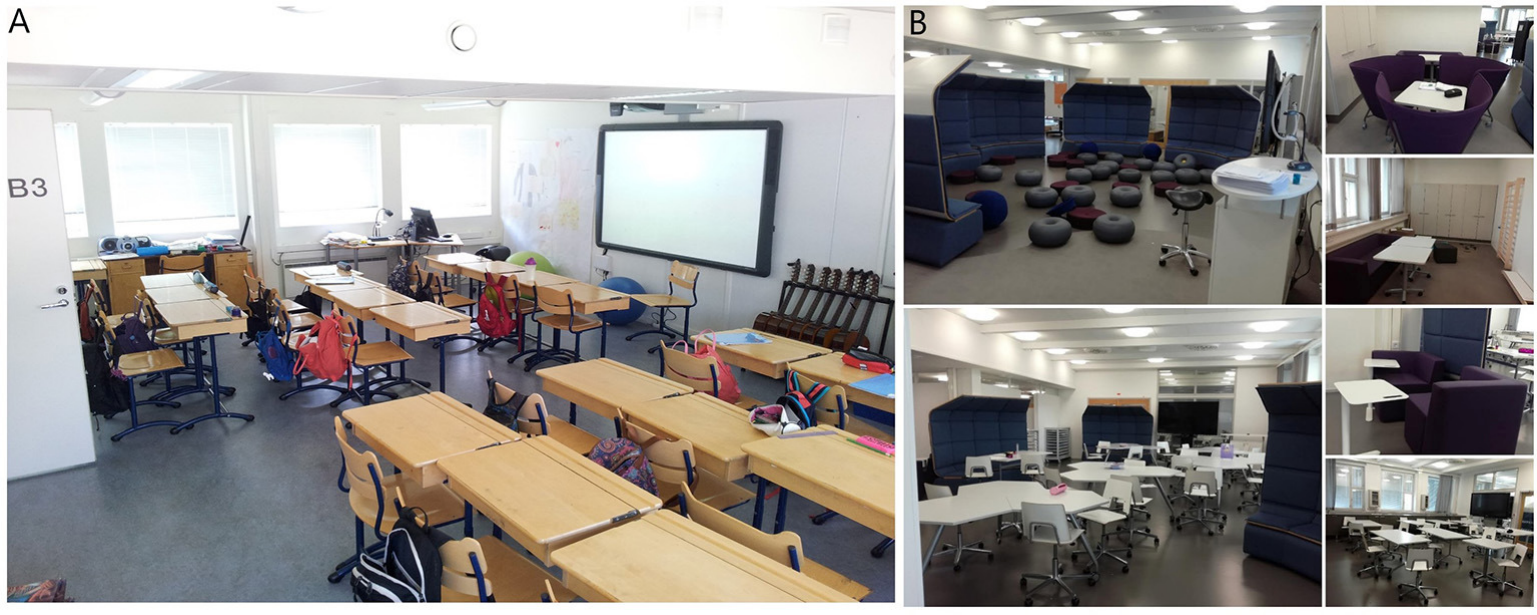
The conventional self-contained classroom **(A)** and the open learning space **(B)**. The pictures from the open learning space show that a one large space has several areas for work allowing division of the class of about 70 students to smaller groups.

The first phase of data collection took place in autumn 2015 in conventional self-contained classrooms with designated desks. The second phase of data collection took place in autumn 2016 when the next cohort of children was studying in the new open learning spaces in the same school after the renovation. The renovated open learning spaces contained a large space with mobile furniture, which afforded multiple options in classroom layout. The instructional area also enabled teaching of arts, physics, and chemistry, while lessons for music and handcrafts were held in their own separate learning spaces. The student did not have an assigned place, such as a designated desk, in the open learning spaces (Figure 1).

Before the renovation, each class was taught by their own classroom teachers in their separate conventional classrooms. After the renovation, students of the same grade-level attended instruction in large open learning spaces, all 3rd graders in their own space and all 5th graders in another space. Teachers of one grade-level collaborated to some extent throughout the week and school days. During the measurement weeks contents of instruction followed the curriculum of the grades in question, and instruction was not in any way altered by the researchers.

ST, BST, and PA were measured during school hours of one school week from Monday to Friday by a waist-mounted triaxial accelerometer (Gulf Coast Data Concepts X16-1, Waveland, USA). Data included in the analyses were determined manually for each student based on the teacher reported weekly schedule for the students. Only time spent inside the classroom was included in the analysis. Physical education, recess, and unusual activities, such as fieldtrips, or activities not part of general education curriculum, such as practice of school festival presentation, were excluded based on the teacher's reported schedule. Examples of 3rd grade curriculums in conventional classrooms and open learning space are provided in Table 1. Furthermore, possible absences from school due to illness or short visits to dentist, for example, during school hours were identified from diaries kept by the students and their parents. The data were also visually inspected to ensure that accelerometers were worn as reported by the participants.

**Table 1:** Examples of 3rd grade students' curriculum during measurement week in conventional classrooms and open learning spaces.

**3rd grade curriculum, conventional classrooms, autumn 2015**					
**Time**	**Monday**	**Tuesday**	**Wednesday**	**Thursday**	**Friday**
8:00–8:45	Distribution of accelerometers			General education	General education
8:45–9:30		General education	General education	General education	General education
9:30–10:00	Recess	Recess	Recess	Recess	Recess
10:00–11:45	Physical education, lunch	General education, lunch^*^	General education, lunch^*^	General education, lunch^*^	General education, lunch^*^
11:45–12:15	Recess	Recess	Recess	Recess	Recess
12:15–13:00	General education	General education	General education	General education	General education
13:00–13.45	General education				
Lessons marked with white were included in analysis and dark gray excluded, ^*^Lunch 11:10–11:30 excluded from analysis.					
**3rd** **grade curriculum, open learning spaces, autumn 2016**					
**Time**	**Monday**	**Tuesday**	**Wednesday**	**Thursday**	**Friday**
8:00–8:45	Distribution of accelerometers	General education			General education
8:45–9:30		General education	General education	General education	General education
9:30–10:00	Recess	Recess	Recess	Recess	Recess
10:00–11:45	General Education, Lunch^*^	General education, lunch^*^	General education, lunch^*^	General education, lunch^*^	General education, lunch^*^
11:45–12:15	Recess	Recess	Recess	Recess	Recess
12:15–13:00	General education			Physical education	General education
13:00–13.45	General education				
Lessons marked with white were included in analysis and dark gray excluded, ^*^Lunch 11:00–11:30 excluded from analysis.					

The measurement range of the accelerometer was ±16 g and the sample rate 40 or 50 Hz with a 16-bit A/D conversion. The resultant acceleration of the triaxial accelerometer signal was calculated from$\sqrt{x^{2}+y^{2}+z^{2}}$, where x, y, and z are the measurement sample of the raw acceleration signal in x-, y-, and z-directions. The number of consecutive data points was 40 or 50 and the corresponding epoch length was one second. Mean amplitude deviation (MAD) was calculated from the resultant acceleration in non-overlapping 1-second epochs. MAD is described as the mean distance of data points about the mean of the given epoch

$$\begin{array}{l} MAD=\frac{1}{n}\overset{n}{\underset{i=1}{\sum }}|r_{i\;-}\bar{r}| \end{array}$$

where *n* is the number of samples in the epoch, *r*_*i*_ is the *i*^*th*^ resultant sample within the epoch and $\bar{r}$ is the mean resultant value of the epoch (Aittasalo et al., 2015; Vähä-Ypyä et al., 2015a). The MAD-method used for assessing PA has documented validity and reliability as an accurate method across different accelerometer brands (Aittasalo et al., 2015; Vähä-Ypyä et al., 2015b). Use of universal PA metrics, such as MAD, in the analysis enables comparison and synthetization of results using different accelerometers (Aittasalo et al., 2015). MAD-values were averaged over 15-second intervals, and averaged values were used to examine time spent at different PA-intensities. Cut-offs were determined as follows: light intensity PA (LPA) 16.7 mg, and MVPA 91 mg (Vähä-Ypyä et al., 2015a). Time spent at different PA intensities was calculated as total minutes of measurement week. Time spent at different intensities was normalized to total classroom time. A BST was determined as any interruption in sedentary time lasting at least 1 min (Saunders et al., 2013). BST was operationalized as the number of breaks per 60 min of classroom time.

### Data Analysis

Statistical analyses were carried out using IBM SPSS Statistics 26 –software (IBM corp. Armonk, NY, USA). We used the Shapiro-Wilk Test for assessing the normality of data distribution. For normally distributed variables, we used independent samples *t-*test to compare the average PA and ST of cohorts assessed before and after renovation. 2-Tailed significances below 0.05 were considered statistically significant. For variables that were not normally distributed, we used Mann-Whitey *U*-test to examine differences in ST, BST, and PA between cohorts. Statistical analyses were made separately for 3rd and 5th grade students. Because of the small sample size, we did not perform further comparisons between boys and girls.

## Results

**Figure 2:**
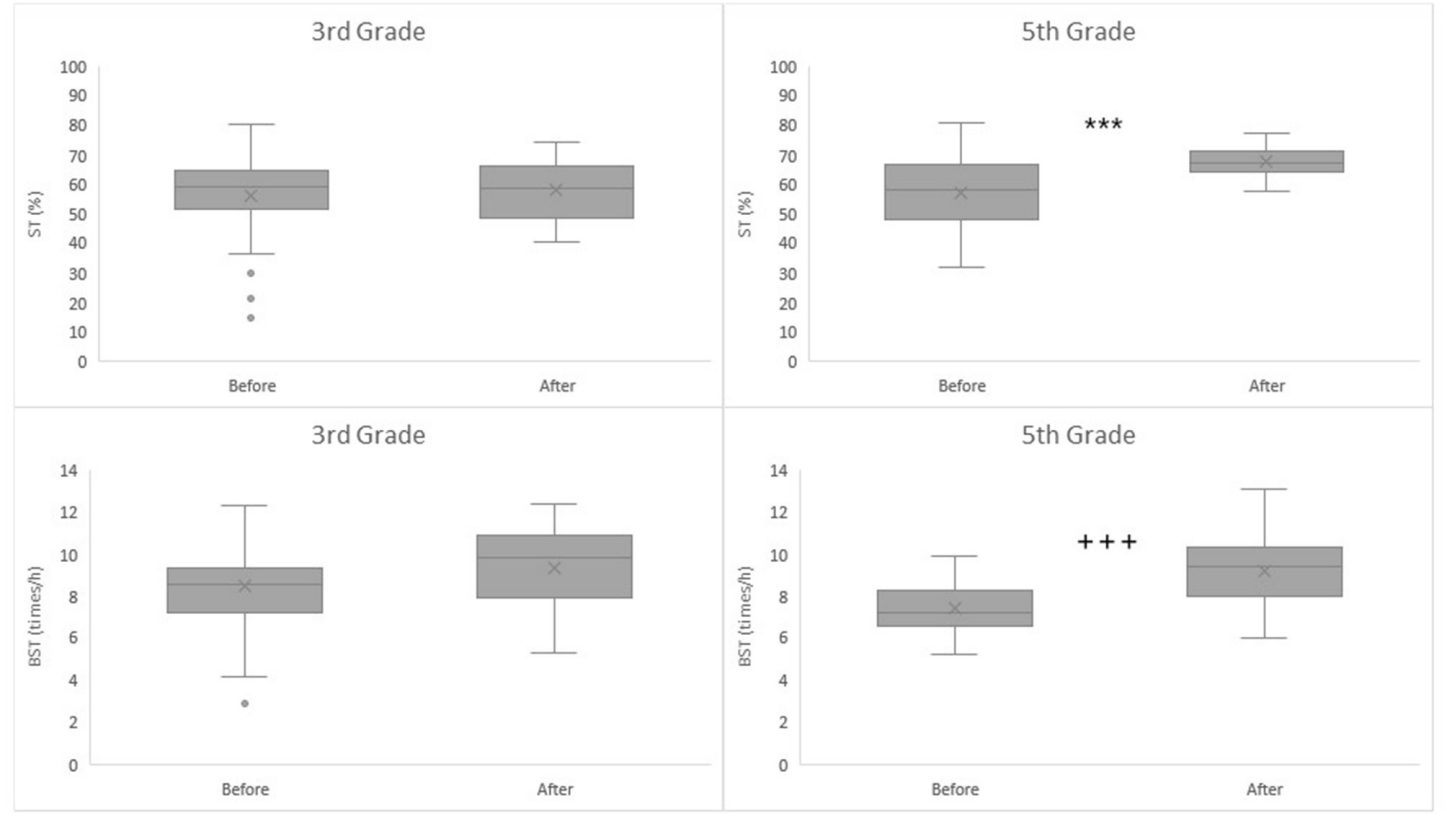
3rd and 5th Grade students' percentages of classroom time spent sedentary (ST) and breaks from sedentary time normalized to 60 min of classroom time (BST) before and after renovation. ^***^Independent samples *t*-test: Before 56.97 ± 12.24%, *n* = 42 vs. After 67.68 ± 5.61%, *n* = 28, mean difference = 10.71%-points, 95%CI = −15.65 to −5.77, *p* < 0.001. Equal variances were not assumed (*F* = 17.642, *p* < 0.001). ^+++^Independent samples *t-*test: Before 7.41 ± 1.16 breaks/h vs. After 9.19 ± 1.59 breaks/h, mean difference = −1.78 breaks/h, 95%CI = −2.486 to −1.079, *p* < 0.001. Equal variances were not assumed (*F* = 2,776, *p* = 0.100).

ST was lower in the cohort of 5th graders assessed before renovation in conventional classrooms than in the cohort of 5th graders measured after renovation in the open learning space. The mean difference of 10.71%-points between cohorts was considered as statistically significant with independent samples *t-*test [95%CI−15.65 to −5.77; *t*_(61.621)_ = −4,945, *p* < 0.001]. Levene's test indicated unequal variances between cohorts, so degrees of freedom were adjusted accordingly. For 3rd graders, significant differences were not observed in ST between cohorts assessed before or after renovation (Table 2, Figure 2).

**Table 2:** Study cohorts and results for sedentary time and physical activity before and after school renovation.

**Measurement**	**Grade**	**Boys/Girls**	**n**	**Duration (min)**	**ST (%)**	**LPA (%)**	**MVPA (%)**	**BST (times/h)**
Before	3rd	19/22	41	792.44 ± 152.60	55.92 ± 14.00	31.55 ± 11.04	11.18 ± 4.56	8.48 ± 2.03
	5th	16/26	42	759.88 ± 180.32	56.97 ± 12.24	28.66 ± 9.99	12.91 ± 7.10	7.41 ± 1.16
After	3rd	11/8	19	609.21 ± 125.03	58.04 ± 10.59	30.01 ± 4.77	14.89 ± 6.43	9.30 ± 1.87
	5th	7/21	28	776.07 ± 201.08	67.68 ± 5.61	22.56 ± 4.59	10.53 ± 2.99	9.19 ± 1.59

*Total classroom of time included in analysis in minutes (Duration), percentages of sedentary time (ST), light physical activity (LPA), and moderate-to-vigorous physical activity (MVPA) from total classroom time. Breaks from sedentary time (BST) normalized to 60 min of classroom time (times/h). Values expressed are means and standard deviations*.

The cohort of 5th grade students assessed before renovation had a smaller number of BST than the cohort measured after renovation. The mean difference of −1.78 breaks/h was considered statistically significant with independent samples *t-*test [95%CI −2.486 to −1.079; *t*_(45.768)_ = −5.100; *p* < 0.001]. Levene's test indicated unequal variances between cohorts and degrees of freedom were adjusted accordingly. For 3rd graders, significant differences were not observed between the cohort assessed before or after the renovation (Table 2, Figure 2).

**Figure 3:**
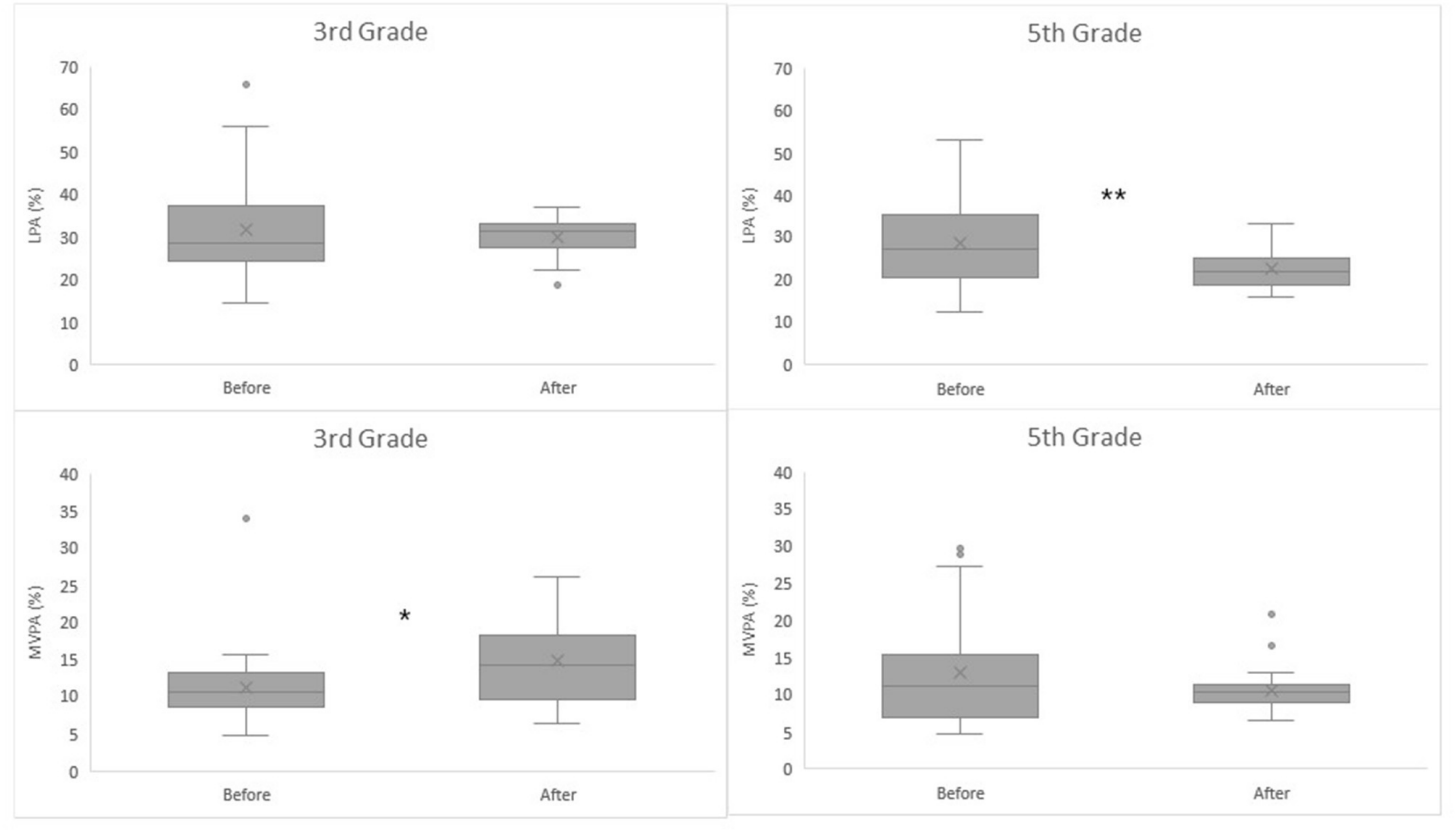
3rd and 5th Grade students' percentages of Light (LPA) and moderate-to-vigorous (MVPA) physical activity from total classroom time before and after renovation. ^**^Independent samples *t-*test: Before 28.66 ± 9.99% vs. After 22.56 ± 4.59%, mean difference = 6.10%, 95%CI = 2.56 to 9.64, *p* = 0.001. Equal variances were not assumed (*F* = 14.618, *p* < 0.001). ^*^A Mann-Whitney-test: Mean Rank (Before) = 27.22, Mean Rank (After) = 37.58, *U* = 524.0, *p* = 0.033.

In the cohort of 5th grade students assessed before the renovation, the average LPA duration was greater than in the cohort measured after the renovation. The mean difference of 6.10 %-points between cohorts was considered statistically significant with independent samples *t-*test [95%CI 2.563 to 9.636; *t*_(61, 655)_ = 3,019, *p* = 0.001]. The Levene's test indicated unequal variances between cohorts and degrees of freedom were adjusted accordingly. In 3rd grade students, MVPA was lower in the cohort measured before renovation than after. A Mann-Whitney-test indicated that the difference was significant [Mean Rank (Before) = 27.22, Mean Rank (After) = 37.58, *U* = 524.0, *p* = 0.033] (Table 2, Figure 3).

## Discussion

We investigated differences in classroom-based ST, BST, and PA in 3rd and 5th grade students before and after a school renovation from conventional classrooms to open learning spaces. We found that the cohort of 5th grade students assessed after renovation in the open learning space had a higher ST, more BST, and less LPA than the cohort measured before renovation in conventional classrooms. In cohorts of 3rd grade students, MVPA was higher after renovation than before.

The results of previous studies have shown that when students are shift to an activity permissive environment and activity permissive teaching methods are used, students ST decreases, number of BST increases, and PA time increases (Lanningham-Foster et al., 2008; Brittin et al., 2017). The current evidence suggests that, elements of flexible learning spaces including a variety of furniture and resources, and a greater use of student-centered pedagogies, facilitate improvements in adolescents' sedentary profiles during class time (Kariippanon et al., 2019). In our present study, we did not observe the expected benefits of reduced ST and increased PA for 5th grade students, although characteristics of an activity permissive classroom are similar to open learning spaces and flexible classrooms (Brittin et al., 2015; Saltmarsh et al., 2015; Kariippanon et al., 2019).

The contrast to findings in previous studies reporting a decrease of ST may be related to differences in study design, such as the inclusion of SB and PA in an outdoor environment (Lanningham-Foster et al., 2008; Brittin et al., 2017). In contrast to many previous studies, the present study examined only indoor classroom PA and SB whereas previous studies have also included recess times. Higher ST observed in open learning spaces may be related to teaching methods used by the teachers. For instance, if two lessons for separate student groups are held simultaneously in the same large learning space, the teachers may need to restrict students' movement to create a quiet learning setting (Michael et al., 2019). Organizing learning in open learning may create barriers for promotion of PA during lessons. These barriers may include institutional factors such as administrative support, availability of resources or lack of time devoted for movement integration, and personal factors such as training and motivation for movement integration, implementation challenges and personal perceptions of value of PA (Michael et al., 2019).

It should be noted that only the 5th graders showed statistically significant differences in ST and LPA, whereas 3rd graders statistically insignificant differences. Our results indicated that among 5th grade students the number of BST was higher after the renovation than before it, which is in line with previous findings (Brittin et al., 2017). Thus, open learning spaces may facilitate short activity bursts during lessons especially among 5th graders.

The cohort of 3rd grade students assessed after the renovation had higher levels of MVPA than the cohort assessed before the renovation. Therefore, our results suggest that younger students may benefit more from open learning spaces than 5th graders. However, typically only a short durations of higher intensity activities are measured during the in-class time, and any increases in MVPA may be more related to transitions from classroom to recess activities. Because we used teacher-reported schedules to select data for analysis, some transitions may have been included, for example, in cases where the teacher had ended the lesson before the scheduled time, or started the lesson later allowing more time for movement in the classroom.

The strengths of the present study include a design allowing analysis of differences in ST, BST, and PA before and after a major school environment renovation using accelerometers and the possibility to focus on classroom behavior. The major limitation of our study was that the teaching practices with respect to allowing movement were not assessed, and therefore the role of interactions between differences in physical environment and teacher instructions could not be specified. Future studies should seek to investigate teachers' and students' interaction with respect to promoting classroom-based PA in open learning spaces. It is also possible that an open learning space facilitates activities that accelerometers are unable to detect. These types of activities may include teacher organized activities, which comprises tasks like balancing. Possible inaccurate reporting of schedules by teachers and inaccurate reporting of diaries by students may also affect our analysis. Especially recess transitions in and out of the classroom may have variable amounts of MVPA, and these transitions need to be better monitored in further studies. However, this inaccuracy has likely been stable before and after the renovation. The limitation of our cross-sectional study design is that it does not determine cause and effect, and thus, studies utilizing longitudinal randomized controlled trials are warranted. There are many types of definitions for SB measurements and unfortunately, there is no clear consensus about the most valid methods among researchers (Altenburg and Chinapaw, 2015). Therefore, a direct comparison between the present study and previous studies is challenging. Furthermore, our relatively small sample size limits generalization of results and therefore studies with larger samples are warranted. As boys have been shown to have less ST, and more MVPA than girls during school hours (van Stralen et al., 2014; Salin et al., 2019), it should be studied if there are any gender specific differences in classroom-based PA in different types of learning environments.

## Conclusions

Despite the greater ST found in 5th graders, schools with open learning spaces may facilitate BST or MVPA as observed in the 5th and 3rd grade cohorts in open learning spaces compared to the cohorts in conventional classrooms, respectively. As prior studies have reported successful environmental interventions in reducing SB and increasing PA, when coupled with student centered pedagogies, content and methods of teaching may be potentially more important contributors to the classroom PA than the classroom environment *per se*. Therefore, teachers should be encouraged to promote PA and use of student-centered pedagogies during classroom time to facilitate opportunities for children to be physically active. The potential PA-limiting or -promoting teacher practices in different types of learning environments need further investigation. Furthermore, studies utilizing longitudinal randomized controlled trials are warranted.

## Data Availability Statement

The datasets generated for this study are available on request to the corresponding author.

## Ethics Statement

The studies involving human participants were reviewed and approved by The University of Jyväskylä Ethics Committee. Written informed consent to participate in this study was provided by the participants' legal guardian/next of kin.

## Author Contributions

Study was originally designed by A-MP, EL, and TF. Data was collected by EL and AP. Data preparation was made by JH and TR. JH was responsible for statistical analyses and drafting of the paper. All authors provided support for data interpretation, feedback on drafts, and approved the final manuscript.

## Conflict of Interest

The authors declare that the research was conducted in the absence of any commercial or financial relationships that could be construed as a potential conflict of interest.
